# Reduced voluntary running performance is associated with impaired coordination as a result of muscle satellite cell depletion in adult mice

**DOI:** 10.1186/s13395-015-0065-3

**Published:** 2015-11-16

**Authors:** Janna R. Jackson, Tyler J. Kirby, Christopher S. Fry, Robin L. Cooper, John J. McCarthy, Charlotte A. Peterson, Esther E. Dupont-Versteegden

**Affiliations:** Department of Rehabilitation Sciences, College of Health Sciences, University of Kentucky, Lexington, KY USA; Department of Physiology, College of Medicine, University of Kentucky, Lexington, KY USA; Center for Muscle Biology, University of Kentucky, Lexington, KY USA; Department of Biology, University of Kentucky, Lexington, KY USA; Present address: Department of Nutrition and Metabolism, School of Health Professions, University of Texas Medical Branch, Galveston, TX USA; Present address: Weill Institute for Cell and Molecular Biology, Cornell University, Ithaca, NY USA

**Keywords:** Satellite cells, Wheel-running, Muscle spindles, Aerobic capacity

## Abstract

**Background:**

Satellite cells, or muscle stem cells, have been thought to be responsible for all muscle plasticity, but recent studies using genetically modified mouse models that allow for the conditional ablation of satellite cells have challenged this dogma. Results have confirmed the absolute requirement of satellite cells for muscle regeneration but surprisingly also showed that they are not required for adult muscle growth. While the function of satellite cells in muscle growth and regeneration is becoming better defined, their role in the response to aerobic activity remains largely unexplored. The purpose of the current study was to assess the involvement of satellite cells in response to aerobic exercise by evaluating the effect of satellite cell depletion on wheel running performance.

**Results:**

Four-month-old female Pax7/DTA mice (*n* = 8–12 per group) were satellite cell depleted via tamoxifen administration; at 6 months of age, mice either remained sedentary or were provided with running wheels for 8 weeks. Plantaris muscles were significantly depleted of Pax7+cells (≥90 % depleted), and 8 weeks of wheel running did not result in an increase in Pax7+ cells, or in myonuclear accretion. Interestingly, satellite cell-depleted animals ran ~27 % less distance and were 23 % slower than non-depleted animals. Wheel running was associated with elevated succinate dehydrogenase activity, muscle vascularization, lipid accumulation, and a significant shift toward more oxidative myosin heavy chain isoforms, as well as an increase in voltage dependent anion channel abundance, a marker of mitochondrial density. Importantly, these changes were independent of satellite cell content. Interestingly, depletion of Pax7+ cells from intra- as well as extrafusal muscle fibers resulted in atrophy of intrafusal fibers, thickening of muscle spindle-associated extracellular matrix, and a marked reduction of functional outcomes including grip strength, gait fluidity, and balance, which likely contributed to the impaired running performance.

**Conclusions:**

Depletion of Pax7-expressing cells in muscle resulted in reduced voluntary wheel running performance, without affecting markers of aerobic adaptation; however, their absence may perturb proprioception via disruption of muscle spindle fibers resulting in loss of gross motor coordination, indicating that satellite cells have a yet unexplored role in muscle function.

**Electronic supplementary material:**

The online version of this article (doi:10.1186/s13395-015-0065-3) contains supplementary material, which is available to authorized users.

## Background

Satellite cells are myogenic stem cells located between the basal lamina and sarcolemma of muscle fibers. They express the paired box transcription factor, Pax7, both when quiescent and when activated in response to stretch, mechanical load, and/or injury [1]. During periods of increased work load, satellite cells are activated, proliferate, and differentiate to contribute to muscle adaptation and repair. The unique capacity of satellite cells to respond to a variety of stimuli and to self-renew has garnered them a presumed role in all forms of muscle plasticity. This assumption was guided by studies using indirect methods [2–4], or correlative observation [5], to assess satellite cell participation in muscle plasticity. Recently, this dogma has been challenged with the advent of genetic mouse models that allow for direct manipulation of satellite cells [6–9]; specifically, the ability to conditionally ablate Pax7+ cells in mature skeletal muscle has provided more insight into the role of satellite cells in muscle adaptation. Employing these models has confirmed that satellite cells are in fact obligatory for muscle regeneration [6, 7, 9] but appear dispensable for adult muscle hypertrophy [6] and regrowth following atrophy [10], implying that muscle regeneration, hypertrophy, and regrowth have different satellite cell requirements. While the function of satellite cells in muscle growth and regeneration is becoming better defined, their contribution to muscle adaptation in response to aerobic exercise remains largely unexplored.

Voluntary wheel running represents a physiological relevant model of aerobic exercise for mice, given the submaximal and prolonged nature of the activity [11]. To determine the role of satellite cells in voluntary wheel running in mice, one study used X-ray irradiation to interrupt DNA synthesis and thus block satellite cell proliferation prior to wheel running in young male C57BL/6J mice [4]. The investigators concluded that blocking cell proliferation did not prevent voluntary running-induced myosin heavy chain isoform shifts nor did satellite cells appear to be involved in angiogenesis, though the researchers did conclude that satellite cell participation was required for muscle growth [4]. However, irradiation is a non-specific approach that perturbs all proliferating cells, not just satellite cells, confounding interpretation. In the rat plantaris, a correlation was reported between satellite cell number and wheel running performance, suggesting that satellite cell content increases as a function of distance run [12]. Furthermore, in a human exercise training model using non-hypertrophic aerobic interval training, satellite cells increased in a fiber-type specific manner [13]. The authors concluded that satellite cells play a role in non-hypertrophic muscle remodeling in response to aerobic exercise, although their specific function in remodeling is unclear. It has been shown that satellite cell number but not myofiber nuclear number increased with free wheel running in vastus lateralis, gastrocnemius, and soleus muscles of rats [14], indicating that proliferation and fusion of satellite cells are not necessarily linked. Consequently, the goal of the current investigation was to directly evaluate the role of satellite cells in adaptation to prolonged aerobic exercise using the Pax7/DTA mouse model to severely deplete satellite cells in adult skeletal muscle [6, 10, 15, 16] prior to voluntary exercise engagement. We hypothesized that hind limb muscles would show impaired adaptation to aerobic exercise in the absence of satellite cell participation. We specifically investigated the plantaris muscle, which is activated during voluntary wheel running [17].

## Methods

### Animal models

All animal procedures were conducted in accordance with guidelines for the care and use of laboratory animals as approved by the Institutional Animal Care and Use Committee of the University of Kentucky. Mice were housed in a temperature and humidity-controlled room and maintained on a 14:10 h light-dark cycle with food and water ad libitum. We utilized a transgenic Pax7 ^*CreER/CreER*^ × Rosa26 ^*DTA/DTA*^ (Pax7/DTA) mouse model that allows for the conditional depletion of >90 % of satellite cells following tamoxifen treatment as previously described [6, 10, 15, 18]. To control for any potential adverse effects of tamoxifen, the parental strain Pax7^*CreER/CreER*^ (Pax7CreER) was employed as a treatment control. Furthermore, to assess the potential for tamoxifen to induce recombination in the brain, a *Pax7* reporter mouse was generated by crossing the Rosa26^ZsGreen/ZsGreen^ × Pax7^CreER/CreER^ creating a Pax7/ZsGreen mouse in which Pax7+ nuclei express *Zoanthus* sp. Green Fluorescent Protein (ZsGreen) upon tamoxifen-induced recombination [19].

### Experimental design

Adult (4-month old) female Pax7/DTA mice (*n* = 8–12 per group) received either an intraperitoneal injection of tamoxifen at a dose of 2.5 mg/day for five consecutive days or were injected with a vehicle control (15 % ethanol in sunflower seed oil) as described previously [6, 10, 15], followed by an 8 week washout period. At 6 months of age vehicle- or tamoxifen-treated mice were singly housed and randomly divided into two groups which either remained ambulatory or were provided with running wheels. The Pax7CreER mice were used to assess the potential independent effects of tamoxifen on running performance. Furthermore, to assess the potential for tamoxifen to cause recombination in the brain, the Pax7/ZsGreen reporter mouse was utilized [19]. Pax7CreER and Pax7/ZsGreen mice received identical tamoxifen treatment as the Pax7/DTA mice.

### Voluntary wheel running protocol

Female Pax7/DTA or Pax7CreER mice at approximately 6 months of age were housed individually in plastic cages measuring 30.5 × 15.2 × 12.7 cm. The mice had open access to running wheels that were mounted within each cage. Ambulatory controls were singly housed in cages of equal dimensions, but without running wheels. A mechanical counter was used to record wheel rotations and was connected to a desktop computer via ClockLab software (Actimetrics, Wilmette, IL). The software analyzed running speed (km/h), total distance run (km/day), total time run (h/day), peak running rate (counts/min), and running bout length (min). The animals had access to food and water ad libitum and were checked daily for health and wellness. Following the 8-week (Pax7/DTA), or 6-week (Pax7CreER), running period, the animals were sacrificed and their plantaris muscles were dissected, processed, and stored at −80 °C for further analyses as described below. Plantaris muscles were chosen for analysis due to their known activation in rodent wheel running protocols [20] and to directly compare the current study’s results with our previous investigations regarding synergist ablation and 8 weeks of plantaris overload in the Pax7/DTA mouse [15].

### Histochemistry/Immunohistochemistry (IHC)

Plantaris muscles were dissected from surrounding connective tissue, weighed, pinned to a cork block at resting length, covered with a thin layer of Tissue Tek OCT compound (Sakura Finetek, Torrance, CA) and quickly frozen in liquid nitrogen-cooled isopentane and stored at −80 °C prior to sectioning. Muscles were sectioned on a cryostat (MicromHM 525) at 7 μm and either used immediately (succinate dehydrogenase (SDH) and cluster of differentiation 31 (CD31)), or frozen at −20 °C for subsequent analysis. Muscles were immunohistochemically analyzed for Pax7 (satellite cells), dystrophin (sarcolemma), myosin heavy chain (MyHC) isoforms, Oil Red O (lipid content) and DAPI (10nM) (4', 6-diamidino-2-phenylindole, Invitrogen, Carlsbad, CA) to visualize nuclei. Additionally, tibialis anterior (TA) muscles and brain tissue were collected from Pax7/ZsGreen mice, cryosectioned and immunohistochemically analyzed for Pax7 and dystrophin. All images were captured using an Axioimager MI upright fluorescent microscope (Zeiss, Göttingen, Germany), and analyses were performed using AxioVision Rel software (v4.8).

#### Pax7

Pax7 was immunodetected on air-dried frozen sections fixed in 4 % paraformaldehyde (PFA) as described previously [6, 10, 15]. Briefly, following fixation sections underwent an epitope retrieval protocol at 92 °C using sodium citrate buffer (10 mM, pH 6.5). Endogenous peroxidase activity was blocked with 3 % hydrogen peroxide in PBS, followed by incubation with the Mouse-on-Mouse Blocking Reagent (Vector Laboratories, Burlingame, CA). Sections were then incubated in Pax7 primary antibody (Developmental Studies Hybridoma Study Bank, Iowa City, IA) at a 1:100 dilution followed by incubation with a goat anti-mouse biotin-conjugated secondary antibody (1:1000) (Jackson ImmunoResearch, West Grove, PA) and subsequently streptavidin-HRP (1:100) included as part of the Tyramide Signal Amplification kit (TSA) (Invitrogen). TSA-Alexa Fluor 488 or 594 (Invitrogen) was used to visualize antibody-binding. Sections were counterstained with DAPI (10 nm) (Invitrogen) for nuclear detection and mounted with Vectashield fluorescent mounting medium (Vector Laboratories). Pax7+/DAPI+ nuclei were counted and normalized per number of fibers. Brain tissue was collected from mice (*n* = 3 per group per mouse strain analyzed) following intracardiac perfusion with 4 % PFA and then subsequently put through sequential sucrose incubations before being frozen, sectioned (10 μm), and allowed to air dry at room temperature. Pax7 IHC was performed on brain tissue using the same protocol as plantaris muscle sections.

#### Dystrophin/DAPI

Air-dried frozen sections were blocked in Mouse-on-Mouse IgG blocking solution (Vector Laboratories). Immediately following the block dystrophin primary antibody ((1:50) Vector Laboratories)) was added to the sections overnight at 4 °C. Sections were then incubated with Texas Red-conjugated goat anti-mouse secondary antibody (1:200) (Rockland Immunochemicals Inc., Gilbertsville, PA). Lastly, sections were post-fixed in 4 % PFA and stained with DAPI. Myonuclear number was assessed by counting DAPI+ nuclei, within the dystrophin boundary. The data are presented as the myonuclei per fiber. Additionally, the dystrophin boundary around each fiber was traced to assess fiber cross-sectional area (μm^2^) and is reported as the mean fiber cross-sectional area per plantaris muscle.

#### MyHC composition

To evaluate fiber-type distribution, frozen sections were air-dried, fixed in methanol, and then incubated with isoform-specific MyHC antibodies: type I (1:100) (BA.D5), type IIa (SC.71), and type IIb (BF.F3) from Developmental Studies Hybridoma Study Bank (Iowa City, IA) overnight at 4 °C. Following washes in PBS, secondary antibodies were applied to the sections at RT as follows: Gt anti-Ms IgG2b, Alexa Fluor 647 conjugated 2 Ab (1:250) (Invitrogen) Gt anti-Ms IgG1, Alexa Fluor 488 conjugated 2°Ab (1:500) (Invitrogen) Gt anti-Ms IgM, and biotin conjugated 2°Ab (1:150) (Invitrogen). Lastly, the sections were incubated in streptavidin-Texas red (1:150) (Vector) and mounted with Vectashield mounting medium (Vector). MyHC isoform expression was manually assessed from ×20 images and expressed as relative fiber-type frequency to account for any fluctuations in fiber number within muscle cross sections.

#### SDH

SDH content within muscle fibers was visualized following 1-h incubation at 37 °C in the following solution: nitro blue tetrazolium (Sigma) and succinate acid disodium (Sigma) and dissolved in 0.2 M of phosphate-buffered saline (PBS). The reaction was performed in a light protected coplin jar, and sections were then sequentially rinsed with 30 and 60 % acetone before being mounted with aqueous mounting media. Images were captured at ×20 magnification, were manually assessed for SDH staining intensity, and were categorized as positive, weakly positive, or negative fibers (++, +, −). The assessor was blinded to the treatment of the animals.

#### CD31

To quantify capillary density, an antibody recognizing the endothelial cell marker CD31 (BD-Pharmingen, 550274) was applied to sections following fixation in ice-cold (4 °C) acetone. Sections were incubated with a rat anti-mouse CD-31 antibody, followed by α-Rat HRP 2° antibody (Impress Kit, Vector Labs MP-7404) and Alexa Fluor 555 conjugated 2°antibody (1:200) in amplification buffer. Images were captured, and CD31+ events were analyzed using the automated thresholding feature of AxioVision Rel software (v4.8) and are reported normalized per muscle fiber.

#### Oil Red O

Lipid content within muscle fibers was assessed by staining with Oil Red O. Oil Red O was dissolved in 60 % triethyl phosphate. Freshly cut sections were air dried, fixed briefly in 37 % formaldehyde at RT, and rinsed in ddH_2_0 prior to staining. Sections were allowed to incubate in Oil Red O solution for 2 h at RT and were subsequently rinsed and mounted in Vectashield. Lipid droplets were visualized fluorescently, and images were captured and analyzed using the automated thresholding feature of AxioVision Rel software (v4.8). Lipid content is reported as total Oil Red O area as a percent of muscle area measured.

#### WGA

Detection of N-acetyl-d-glucosamine was evaluated on frozen muscle sections using Texas Red-conjugated wheat germ agglutinin (WGA) (eBiosciences, San Diego, CA). Sections were fixed in 4 % PFA and then incubated with WGA conjugate for 2 h at RT. Images were captured at ×10 magnification, and the staining was quantified using the thresholding feature of the AxioVision Rel software. The area occupied by WGA was expressed either relative to muscle fiber number, or in the case of spindle fiber analysis, it was normalized to spindle fiber circumference to account for total spindle size and reported as the extracellular matrix (ECM) index. Furthermore, WGA images were used to trace the cross-sectional area of intrafusal fibers located within spindle fibers. The average cross-sectional area of all intrafusal fibers within a given spindle fiber was averaged and reported as the mean intrafusal fiber area (μm^2^) per spindle fiber.

### Voltage dependent anion channel (VDAC) Western blot

Plantaris muscles were homogenized in radioimmunoprecipitation assay (RIPA) buffer containing protease inhibitor cocktail (Sigma-Aldrich, St. Louis, MO). Protein concentrations were quantified using a BCA (Thermo) assay. Thirty micrograms of protein were separated on a 4–15 % SDS polyacrylamide gel (Bio-Rad, Hercules, CA) and subsequently transferred onto nitrocellulose membranes. Membranes were blocked in Odyssey blocking buffer (LI-COR, Lincoln, NE) and immunoblotted with a VDAC (1:1000) primary antibody (Cell Signaling) overnight at 4 °C. Following incubation with goat anti-rabbit IgG (Alexa 680) (LI-COR) secondary antibody, immunoreactive bands were visualized using the Odyssey Infrared Imaging System. Band intensity was quantified using Odyssey Infrared Imaging System Application Software Version 3.0.21, and results are expressed as arbitrary densitometric values of VDAC normalized to actin to assure equal protein loading.

### Functional outcomes

A battery of functional measurements were performed on 6-month-old female Pax7/DTA mice 8 weeks following either vehicle or tamoxifen treatment (*n* = 10 vehicle, 10 tamoxifen-treated).

#### Grip strength

Forelimb grip strength was measured by allowing mice to grab a bar attached to a force transducer as it was pulled horizontally by the tail away from the bar (Model 1027CSM; Columbus Instrument Co., Columbus, Ohio) [21]. The test was performed five times, and the average peak force (N) for each mouse was normalized to body mass (g) to determine grip strength for each mouse.

#### Gait analysis

The gait of each mouse was evaluated during part of a standard rodent functional observational battery [22]. Gait assessment was given a score of 0 if the mouse exhibited a fluid gait with pelvic elevation and a score of 1 if the mouse presented with an irregular gait and/or an abnormal pelvic tilt. The examiner was blinded to the treatment of the mice.

#### Rota-Rod test

Sensorimotor coordination was assessed using a Rotor-Rod apparatus consisting of a rotating rod suspended 18 in. above a padded floor (San Diego Instruments, San Diego, CA). This system uses a mouse’s natural fear of falling as a motivational tool to test gross motor function. Mice were placed on the rotating rod at a speed of 4 rpm for 60 s for their training sessions (two training sessions separated by at least 10 min). After successful completion of the training sessions and adequate rest, the speed of the rod is gradually increased to a maximum of 40 rpms for each of the three testing sessions. The trial is complete when the animal falls, or the time period ends (300 s max). Latency to fall (s) and distance traveled (cm) were recorded, and the average of the three testing trials was reported for each animal.

#### Balance beam

A beam walking protocol was used to evaluate mice on their ability to traverse beams of decreasing widths. The beam walking protocol evaluates motor balance and coordination by assessing both the time it takes the mice to traverse the beam and the number of foot slips that the mouse experiences during the crossing. This test is more precise in detecting subtle deficits in motor skills and balance that may not be detected by other motor tests, such as the Rota-Rod [23]. Following standard acclimation and training, three beam widths were used to assess balance (28, 17, and 11 mm) and the mice were given 1 min per trial to complete the crossing of the beam to a bedding filled safe-room on the far end of the beam. The mean of all values per beam obtained in the five trials was used for analysis.

#### Muscle spindle activity assays

Extensor digitorum longus (EDL) muscles were chosen for this assay because they have two tendons and nerve endings that can be easily dissected and have similar fiber-type composition as the plantaris muscle. Electrophysiological methods were performed as described and modified as needed [24]. Briefly, EDL muscles with the associated peroneal nerve were carefully dissected from euthanized animals and bathed in Lily’s solution (see [24]). The proximal and distal tendons of the EDL were then double tied with silk thread; one tendon was attached to a stationary position with stainless steel staples and the other to a stainless steel hook attached to a speaker (Model SF-9324, Pasco Scientific, Denmark) driven by a DC source from a stimulator (Grass S88, Grass Products, Natus Neurology). Movement excursions were calibrated by observing the attachment of the thread to the tendon with a millimeter grid placed under the recording dish and used to accurately and reproducibly stretch the muscle 1 mm (10 % of muscle length). A suction electrode made from glass pipettes fitted with plastic tips was used to record extracellular signals from the cut nerve and the extracellular signals were amplified via a P-15 (Grass Products, Natus Neurology). A PowerLab 4SP (AD Instruments) in conjunction with a computer was used for data acquisition at 20 kHz on line. LabChart software (AD Instruments) was used for offline analysis of firing frequency (evoked activity) in response to the stretch. Analysis was analogous to the procedure previously described [24]. The 1-mm stretches were given 10 s apart while the muscle was pulled taut to a set length where background muscle spindle firing was minimal to allow for reproducibility with each stretch. Five stretches of 4-s duration were provided in one series. The last three consistent stretches were used for analysis for consistency. Within each stretch period approximately the last second was used to count the number of spikes recorded above a set threshold over the noise, to ensure that the dynamic response of the muscle spindle had a consistent firing during the static phase. An average firing frequency of the three stretches in the series was calculated per muscle and the average reading of two legs for the same animal is reported (*n* = 2 per treatment group).

### Statistics

Data were analyzed with SigmaPlot software (Systat Software, San Jose, CA) via a two factor ANOVA, or a two factor repeated measures ANOVA (running data). If a significant interaction was detected, an applicable post hoc analysis was employed to determine the source of the significance. Additionally, non-paired Student’s *t* tests were used where appropriate. Statistical significance was accepted at *P* ≤ 0.05. Data are reported as mean ± standard error of the mean.

## Results

### Wheel running did not affect Pax7+ cell number or myonuclear counts

**Fig. 1:**
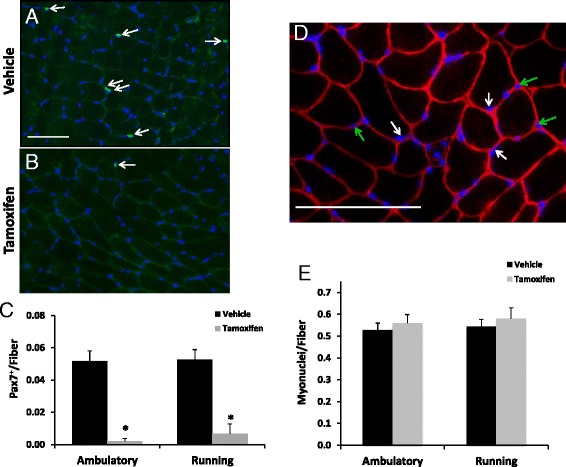
No myonuclear accretion with wheel-running in adult mouse plantaris muscles irrespective of satellite cell depletion. **a–b** Pax7 immunohistochemistry was performed on plantaris muscle cross sections (7 μm) from vehicle (**a**)- and tamoxifen (**b**)-treated animals to detect the presence of satellite cells (*green*). Sections were counterstained with DAPI (*blue*) and Pax7+/DAPI+ nuclei were quantified (*white arrowheads*). Scale bar = 100 μm. **c** Quantification of Pax7+/DAPI+ nuclei. Data are represented as Pax7+ nuclei per fiber. (**d**) Myonuclear number was assessed in plantaris muscle cross sections that were immunohistochemically analyzed for dystrophin (*red*) and co-stained with DAPI (*blue*). DAPI+ nuclei residing within the dystrophin boundary are classified as myonuclei (*white arrows*). Interstitial nuclei are denoted with *green arrows*. A representative image from an ambulatory vehicle muscle is shown. Scale bar = 50 μm. **e** Quantification of myonuclei represented as myonuclei per muscle fiber. *Asterisk* indicates a significant effect of tamoxifen between condition-matched groups. All values are presented as mean ± SE. Significance was set at *p* ≤ 0.05

The efficiency of tamoxifen-induced satellite cell depletion was measured by IHC in the plantaris muscles from both sedentary and wheel running Pax7/DTA mice. Pax7+/DAPI+ cells were identified (white arrows, Fig. 1a, b) and quantified (Fig. 1c), and the results demonstrated effective satellite cell reduction (~90 %; *P* < 0.05) in tamoxifen-treated mice. Furthermore, there was no increase in Pax7+ cells following 8 weeks of voluntary wheel running in vehicle- or tamoxifen-treated animals, revealing that tamoxifen-treated mice with running wheels remained effectively depleted of satellite cells and furthermore that satellite cells were not increased following 8 weeks of wheel running in mice with a full complement of satellite cells (Fig. 1a–c). Additionally, myonuclear number was assessed by quantifying DAPI positive nuclei within the dystrophin border (Fig. 1d, e) and reported as number of myonuclei per fiber (Fig. 1e). A representative image from an ambulatory vehicle muscle is shown (Fig. 1d). Myonuclear number was not changed by wheel running and was not affected by satellite cell depletion.

### Diminished running capacity in satellite cell-depleted mice

**Fig. 2:**
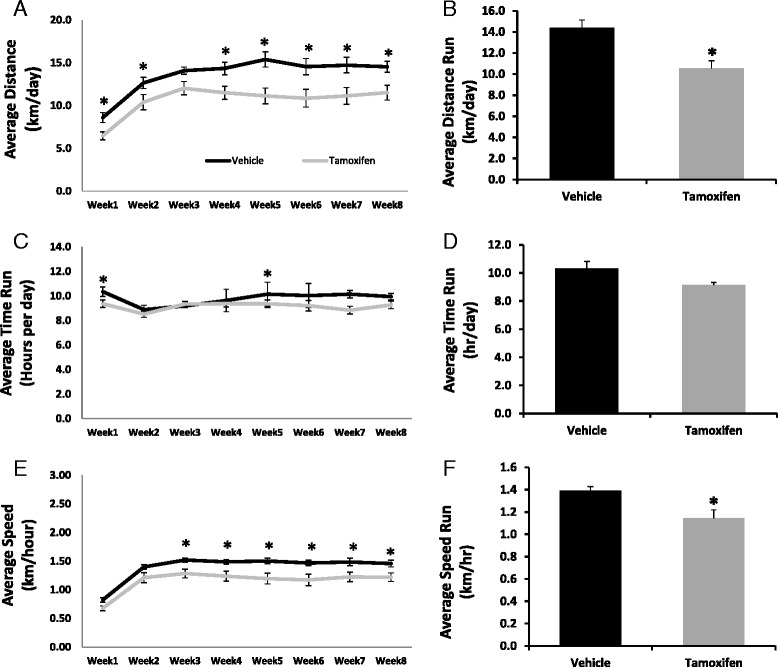
Satellite cell depletion results in reduced running performance. To assess running performance, mice were given access to cage-mounted running wheels with mechanical counters attached to a computer for 8 weeks. **a** Average distance (km/day) run per week over 8 weeks. **b** Average distance (km/day) run over the entire 8-week wheel running protocol. **c** Average time (h/day) run per week over 8 weeks. **d** Average time (h/day) run over the entire 8-week wheel running protocol. **e** Average speed (km/h) run per week over 8 weeks. **f** Average speed (km/h) run over the entire 8-week wheel running protocol. Values are presented as mean ± S.E. *Asterisk* indicates a significant effect of tamoxifen. Significance was set at p ≤ 0.05

Vehicle-treated Pax7/DTA mice ran on average 26.8 % (*P* < 0.05) more kilometers per day than their tamoxifen-treated counter parts averaging 14.4 km/day compared to 10.5 km/day, respectively (Fig. 2a, b). Likewise, satellite cell-depleted animals ran ~18 % (*P* < 0.05) slower than non-depleted animals (1.15 ± 0.07 vs. 1.39 ± 0.03 km/h) (Fig. 2e, f). However, there was no statistically significant difference in the average amount of time spent per day running (Fig. 2d), although at week 1 and week 5, the hours run per day were significantly different between control and satellite cell-depleted animals (Fig. 2c). Interestingly, with regard to both distance and speed, satellite cell-depleted mice already showed deficiencies in running performance at the start of the protocol, and these depressed levels remained relatively constant throughout the remainder of the running protocol (Fig. 2a, e). Although the ability to capture actual maximal speed is not possible using our experimental set up, we were able to assess the peak rate at which the mice could turn over the running wheel (counts per minute). Peak rates of wheel rotation were significantly lower in satellite cell-depleted mice (Additional file 1 A). Furthermore, when the duration of running was broken down into bouts (defined as periods of running with more than an 18-min break between them), satellite cell-depleted mice tended to run for shorter bouts than vehicle-treated mice (Additional file 1 B). Moreover, it should be noted that running mice maintained their body weight over 8 weeks of wheel running only losing on average 1 % of total body weight irrespective of tamoxifen treatment (data not shown).

Lastly, to assure that tamoxifen was not having a toxic effect on the mice independent of satellite cell depletion, the parental strain, Pax7CreER, was used as a treatment control and underwent the identical tamoxifen treatment regime as the Pax7/DTA mice followed by 6 weeks of voluntary wheel running. There was no difference in the distance run between vehicle and tamoxifen-treated Pax7CreER mice (Additional file 2). Moreover, when the hearts from these mice were weighed immediately following sacrifice, there was no difference in heart weights (mg), or heart weights normalized to body weight (mg/g) (data not shown). These data indicate that it is the loss of Pax7+ cells that results in lower running capacity and not a side effect of tamoxifen treatment or Cre toxicity.

### MyHC distribution and markers of metabolic adaptation were altered following 8 weeks of wheel running independent of satellite cell content

**Fig. 3:**
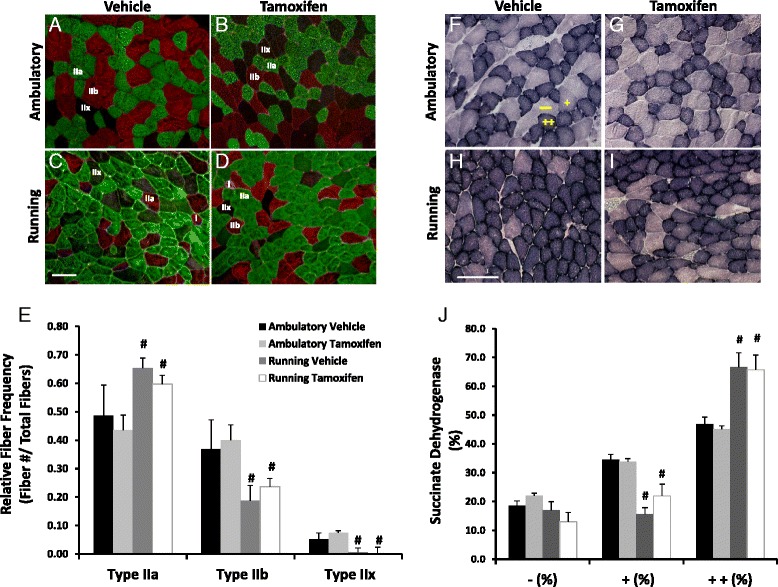
Eight weeks of voluntary wheel running resulted in a shift in myosin heavy chain isoform distribution and an increase in SDH staining in mouse plantaris muscles, independent of satellite cell depletion. **a**–**d** Representative images of plantaris muscle cross sections were examined immunohistochemically for myosin heavy chain myosin (MyHC) type IIa (*green*), MyHC IIb (*red*), and MyHC IIx (*unstained/black*). Scale bar = 100 μm. **e** Quantification of relative frequency of MyHC expression. Data are presented as mean relative frequency ± S.E.M. **f**–**i** Representative ×20 images of SDH staining from each experimental group. **j** Quantification of SDH positive fibers as a percent of total fibers; (−) negative fibers, (+) weakly positive staining fibers, (++) strongly positive staining fibers. Values are mean ± SE. *Number sign* indicates a significant difference between treatment-matched ambulatory and running animals. Significance was set at *p* ≤ 0.05

To investigate potential mechanisms underlying the altered running behavior of the satellite cell-depleted Pax7/DTA mice, muscle fiber-type differences and changes in muscle metabolic markers were assessed. Plantaris muscles from running mice exhibited an 18 % reduction in fast-twitch glycolytic fibers (MyHC IIb) and a 17 % increase in fast-twitch oxidative fibers (MyHC IIa). Furthermore, MyHC IIx fibers were almost completely absent in wheel-run mice (Fig. 3a–e). This shift to a more oxidative MyHC phenotype following voluntary wheel running was unaffected by satellite cell depletion. Correspondingly, SDH activity was evaluated as an estimate of oxidative capacity in both ambulatory and wheel running mice. Consistent with the shift in fiber-type distribution, SDH staining intensity significantly increased with running resulting in >20 % increase in strongly positive fibers compared to treatment-matched ambulatory animals irrespective of satellite cell depletion (Fig. 3f–j).

**Fig. 4:**
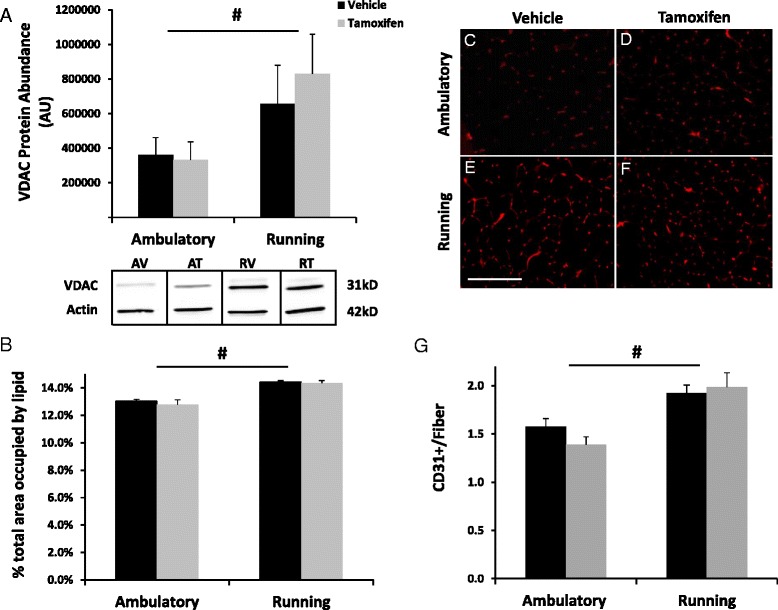
Mitochondrial content, muscle fiber vascularization, and intracellular lipid aggregates were increased with running and unaffected by satellite cell depletion. **a** Voltage dependent anion channel (VDAC) was evaluated via immunoblotting as a rough estimate of mitochondrial content. The density of the resulting immunopositive bands was quantified and normalized to actin as a loading control (arbitrary units). **b** Quantification of Oil Red O staining performed on plantaris muscle cross sections to depict muscle lipid droplets. **c**–**f** CD31 was used as an endothelial marker to estimate muscle fiber vascularization. Representative images from each experimental group. Scale bar = 100 μm. **g** Quantification of CD31^+^ events was performed using an automated thresholding program and is reported as CD31^+^ events per muscle fiber. *Number sign* denotes main effect of running. Data are presented as means ± SE, with significance set at *p* ≤ 0.05

To further assess the effect of satellite cells on aerobic adaptations to wheel running, VDAC abundance was assessed as an estimate of mitochondrial density. VDAC content demonstrated a more than twofold increase in plantaris muscles from wheel running mice compared to their ambulatory counterparts, but this increase was unaffected by satellite cell depletion (Fig. 4a). As an index of the potential for substrate storage in the form of intramuscular triglycerides, Oil Red O staining was employed to assess the presence of intramyocellular lipid droplets. The total area occupied by lipid within plantaris muscle cross sections was visualized fluorescently and found to be modestly, but significantly increased with wheel running (Fig. 4b); however, lipid content was not influenced by satellite cell depletion. Lastly, CD31 was used as an endothelial cell marker to estimate muscle fiber vascularization to assess whether or not satellite cell depletion affected the increase in capillary density that is observed in skeletal muscles from wheel running mice [25]. Eight weeks of wheel running resulted in a 32 % increase in capillary-to-fiber ratio in plantaris muscles irrespective of satellite cell depletion (Fig. 4g).

### Extracellular matrix content and plantaris muscle fiber size remained unaltered following voluntary wheel running

**Fig. 5:**
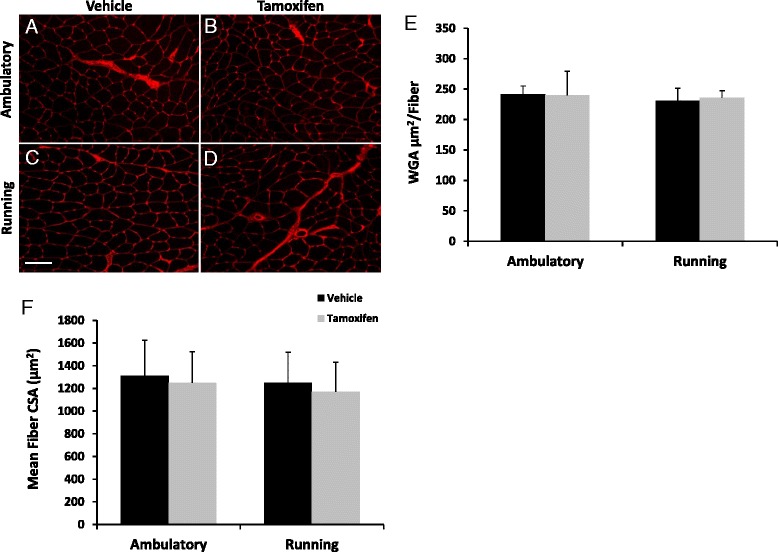
Extracellular matrix deposition and plantaris fiber size remained constant following 8 weeks of wheel running irrespective of satellite cell ablation. **a–d** Plantaris muscle cross sections were histochemically stained for WGA (binds N-acetyl-d-glucosamine; *red*). Scale bar = 50 μm. **e** Quantification of area occupied by WGA normalized to muscle fiber. **f** Mean plantaris muscle fiber cross-sectional area (CSA) was quantified by tracing dystrophin-labeled fibers (not shown). Values are means ± SE. Significance was set at *p* ≤ 0.05

Excess extracellular matrix deposition is associated with inhibited long-term muscle hypertrophy in plantaris muscles from Pax7/DTA mice following mechanical overload [15]. WGA, an estimate of extracellular matrix deposition, was quantified as an estimate of fibrosis. WGA remained constant across all experimental conditions (Fig. 5a–e), suggesting that there is no increase in fibrosis with wheel running or satellite cell depletion in adult female Pax7/DTA mice. Similarly, there were no changes in plantaris muscle fiber cross-sectional area (μm^2^) attributed to wheel running and/or satellite cell depletion (Fig. 5f), indicating that wheel running does not elicit a hypertrophic stimulus in female mouse plantaris muscles. The mean plantaris fiber cross-sectional area data are congruent with the plantaris muscle wet weight data which remained unchanged with wheel running (Additional file 3).

### No tamoxifen-induced recombination in the brain

**Fig. 6:**
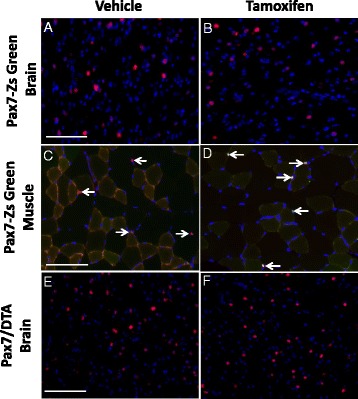
No detectable Cre recombination or depletion of pax7+ nuclei in response to tamoxifen in brain tissue. To verify that tamoxifen was not depleting Pax7+ cells in the brain, and consequently causing centrally mediated effects, Pax7-ZsGreen reporter mice and Pax7/DTA mice were treated with tamoxifen and assessed for either the presence of ZsGreen positive nuclei or a depletion of Pax7+ nuclei, respectively. Representative images of brain (**a–b**) and tibialis anterior muscles (**c–d**) from the same ZsGreen animals were immunohistochemically probed for Pax7 (*red nuclei*). *White arrows* indicate Pax7+ nuclei in tibialis anterior muscles from both vehicle-treated (**c**) and tamoxifen-treated animals (**d**). Tamoxifen-treated muscles have GFP+ labeled Pax7 + cells (red/green overlay in **d**) indicating tamoxifen-induced recombination which is notably absent in both the vehicle-treated ZsGreen muscle and in both the vehicle (**a**)- and tamoxifen (**b**)-treated ZsGreen brain sections. Representative images of brain from Pax7/DTA mice (**e**–**f**) immunohistochemically probed for Pax7 (*red nuclei*). Scale bars = 100 μm

To assess the potential for tamoxifen to cause recombination in Pax7-expressing cells in the brain and introduce an unintended central nervous system component into our experimental model by depleting Pax7+ cells in brain tissue, a Pax7/ZsGreen reporter mouse was utilized. In these mice, Pax7-containing nuclei will express ZsGreen upon tamoxifen administration via Pax7-driven Cre-dependent recombination using the same Rosa26 locus as the Pax7/DTA mouse, thus assuring comparable recombination efficiencies. Brains and TA muscles from Pax7/ZsGreen mice treated with tamoxifen and a vehicle control were evaluated both microscopically (for presence of ZsGreen caused by tamoxifen-induced recombination) and immunohistochemically for the presence of Pax7. Pax7+ nuclei in TA muscles from both vehicle-treated and tamoxifen-treated animals are marked with white arrows (Fig. 6c, d). Tamoxifen-treated muscles have ZsGreen^+^ labeled Pax7+ cells (red/green overlay) as shown with white arrows in Fig. 6d, indicating tamoxifen-induced recombination in TA muscle. This co-label of ZsGreen and Pax7 was notably absent in vehicle-treated muscle and in both vehicle- and tamoxifen-treated brain sections (Fig. 6a–c) indicating that recombination had occurred in muscle tissue, but not in the brain. Additionally, brains sections from Pax7/DTA mice were immunohistochemically analyzed for the presence of Pax7+ cells. Congruent with the findings in the Pax7/ZsGreen reporter mice, no Pax7+ depletion occurred, indicating that tamoxifen does not induce Cre-loxP recombination in the brain, using our protocol. Therefore, we suggest that the effects of Pax7+ cell ablation on running performance are not centrally mediated, but rather muscle-specific.

### Satellite cell depletion resulted in gross motor functional deficits

**Fig. 7:**
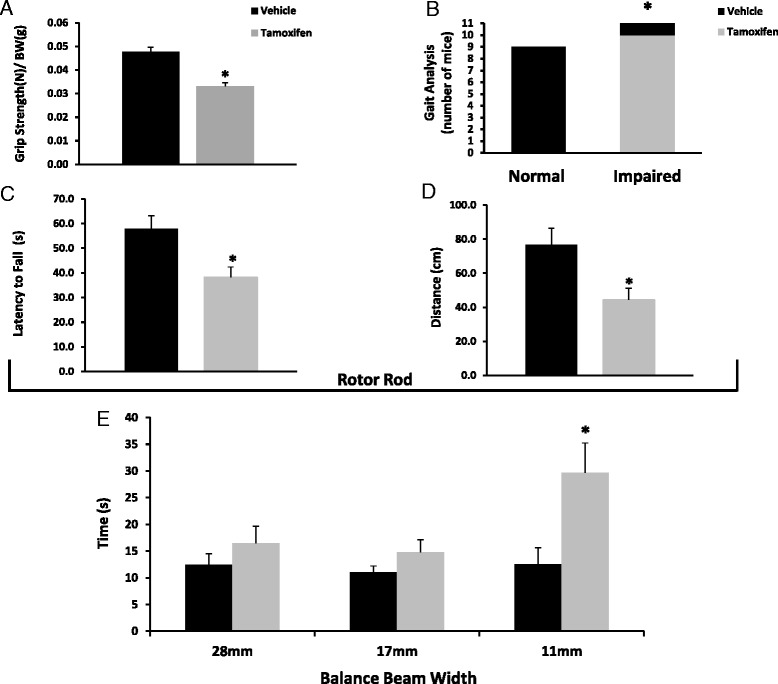
Satellite cell depletion resulted in a pronounced reduction in functional outcomes pertaining to strength, coordination, and balance. To assess functional measures beyond running performance, vehicle- and tamoxifen-treated adult female Pax7/DTA mice were subjected to a battery of functional tests. **a** Grip strength was measured using a specialized force transducer and reported as (N/g of body weight). **b** Gait analysis was performed as part of a standard observational battery. Vehicle- and tamoxifen-treated animals were separated into two categories those with normal gaits or impaired gaits. The two experimental conditions were evaluated using a chi-squared test. **c**–**d** A Rotor-Rod apparatus was used to evaluate motor coordination and balance. The results are reported as latency to fall (s) (**c**) and distance traveled on the rod (cm) (**d**). **e** Balance beams of varying widths were used to specifically assess balance, with total time to traverse each beam (seconds) as an endpoint measurement. *Asterisk* indicates significant difference between vehicle and tamoxifen. Values are means ± SE. Significance was set at *p* ≤ 0.05

In order to test whether the decrease in running activity in satellite cell-depleted mice is due to functional deficits, a battery of tests was performed. Grip strength, gait fluidity, and balance, as measured by both a balance beam test and a rotating rod trial, were evaluated in 6-month-old female Pax7/DTA mice 8 weeks following either vehicle, or tamoxifen treatment (*n* = 10 per group). Satellite cell-depleted animals showed marked decrements in grip strength, only obtaining ~60 % of the force production recorded in non-depleted mice (Fig. 7a). Furthermore, a standard rodent observational battery showed that all of the satellite cell-depleted animals (*n* = 10) exhibited abnormal gaits classified as irregular, including an abnormal pelvic tilt compared to only one out of 10 vehicle-treated animals (Fig. 7b). To further examine balance and coordination, mice were evaluated on a Rotor-Rod. Tamoxifen-treated animals underperformed in both the time they were able to stay on the rod(s) and the distance traveled (cm) when compared to their vehicle-treated counterparts. On average, satellite cell-depleted animals fell from the rods 34 % sooner and covered 40 % less distance when compared to control animals during the trials (Fig. 7c, d). As a final and more sensitive test to assess balance and coordination decrements, mice were tested on their ability to traverse balance beams of varying widths (28, 17, and 11 mm). Satellite cell-depleted mice took significantly longer to cross the narrowest beam compared to non-depleted mice (29.7 ± 5.5 vs. 12.5 ± 3.1 s, respectively) (Fig. 7e). Results from these functional assessments indicate the existence of functional locomotor deficits in satellite cell-depleted animals that are similar to those observed in mice with proprioception insufficiency [26].

### Depletion of Pax+ cells induces morphological and functional changes in muscle spindles

**Fig. 8:**
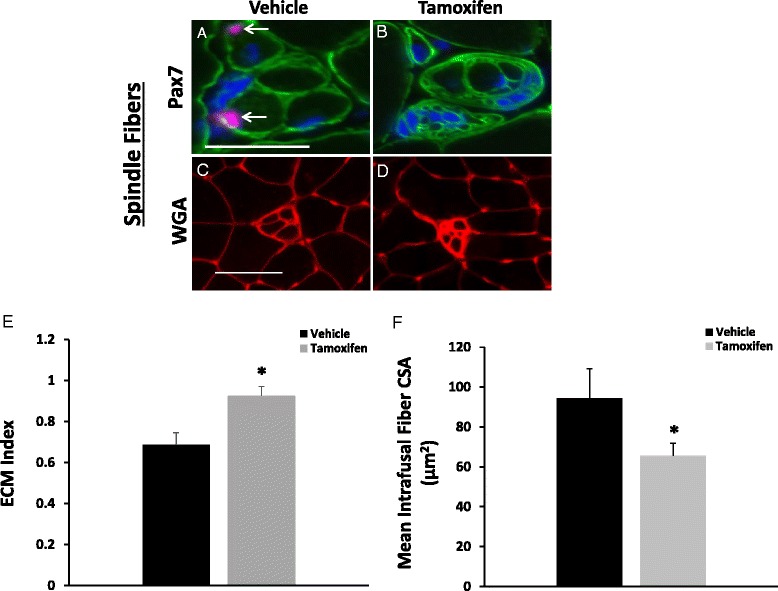
Increased extracellular matrix and decreased intrafusal fiber cross-sectional area in Pax7+ cell depleted mice. Representative images of muscle spindle fibers from plantaris muscles of vehicle (**a** and **c**)- and tamoxifen-treated (**b** and **d**) mice. Plantaris muscle cross sections were examined immunohistochemically for Pax7 (*red*), laminin (*green*), and counterstained with DAPI (*blue*) (**a**–**b**). Representative images of spindle fibers stained with WGA (**c** and **d**). Scale bars represent 50 μm. Quantification of area occupied by WGA within spindle capsules and enclosed intrafusal fibers normalized to muscle spindle circumference (**e**). Values are reported as the extracellular matrix (ECM) index. Mean intrafusal fiber cross-sectional area (μm^2^) within individual muscle spindles was quantified by tracing WGA-stained intrafusal fibers (**f**). *Asterisk* indicates significant difference between vehicle and tamoxifen. Values are means ± SE. Significance was set at *p* ≤ 0.05

**Fig. 9:**
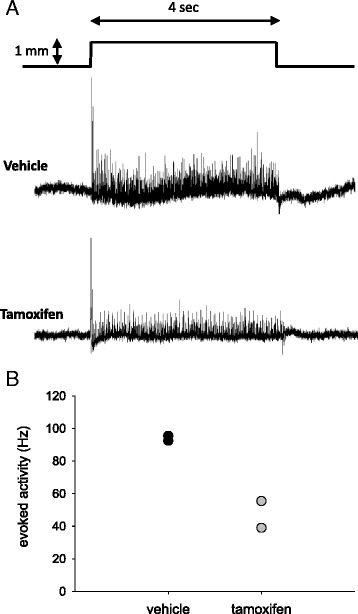
Reduced firing frequency in response to stretch of muscle spindles from satellite-depleted animals. Representative tracings from EDL muscle preparations from both vehicle- and tamoxifen-treated animals (*n* = 2) in response to a 1-mm stretch (**a**). Quantification of firing frequency reported as evoked activity (Hz) (**b**). *Individual dots* indicate data averaged between two legs for each animal

In addition to the depletion of satellite cells from extrafusal fibers, Pax7+ cells were depleted from the intrafusal fibers in muscle spindles of tamoxifen-treated Pax7/DTA mice (Fig. 8a, b). Muscle spindles lacking Pax7+ cells also appeared less organized (Fig. 8b, d). ECM deposition both in the spindle capsule and surrounding the intrafusal fibers within each muscle spindle was more abundant in satellite cell-depleted animals than their vehicle control counterparts (Fig. 8c–e). Moreover, mean intrafusal fiber CSA (μm^2^) within individual muscle spindles was significantly reduced by 30 % in satellite cell-depleted mice (Fig. 8f) signifying a potential role of satellite cells in mediating intrafusal fiber maintenance. In addition, electrophysiological recordings from satellite cell-depleted mice showed reduced firing frequencies (evoked activity in Hz) in response to stretch compared to muscles from mice with a full complement of satellite cells (Fig. 9a, b).

## Discussion

The purpose of the present study was to investigate the role of satellite cells during prolonged aerobic exercise. We hypothesized that satellite cell depletion would impair muscle adaptation to wheel running in hind limb muscles. Our results indicate that satellite cell depletion is detrimental to both wheel running performance and gross motor coordination, but intrinsic adaptations in muscle properties normally associated with aerobic exercise were not affected.

It has long been dogma that skeletal muscle plasticity, irrespective of the stimulus, is directly tied to the action of satellite cells on existing myofibers; recent studies indicate that is only be partially true. Satellite cells are indeed obligatory for tissue regeneration and repair in response to injury [7, 9]; however, they are not required for acute muscle hypertrophy [6] or regrowth following an atrophic stimulus [10]. Furthermore, recent work from our laboratory [27] and others [28] demonstrated that despite living most of their adult life without satellite cells, there was no observable phenotypic change in aged Pax7/DTA mice that were satellite cell depleted at 4 months of age leading to the conclusion that satellite cells are not required for normal muscle maintenance across the adult mouse lifespan. Even though these studies question the requirement of satellite cells for adult muscle adaptation and maintenance, it is currently unclear whether they play a role in aerobic exercise involving a non-loading and non-injurious stimulus.

Wheel running utilized in this study is a non-stressful, voluntary behavior [11] and is therefore an effective physiological method to aerobically challenge rodents. Its efficacy is confirmed by indices of aerobic capacity and mitochondrial biogenesis such as citrate synthase [12] and succinate dehydrogenase activity [29]. Furthermore, voluntary wheel running elicits MyHC isoform fiber-type transitions [4, 29, 30] and increased muscle capillarization [4, 25] that are well-documented adaptations to aerobic exercise. We show that wheel running was indeed associated with increased SDH staining, elevated muscle vascularization, a shift in MyHC to more oxidative isoforms, as well as higher VDAC protein abundance and lipid accumulation. Concomitantly, no change in muscle size was observed with wheel running, consistent with previous reports [12, 30–32], indicating that our wheel running model was effective in aerobically training the mice without hypertrophy.

The role of satellite cells in aerobic muscle adaptation has mainly been addressed through correlational observations in rodents and humans [12, 13, 33]. Although some studies have shown increases in satellite cell content following voluntary wheel running [4, 12], we did not observe an increase in satellite cell number with wheel running in our vehicle-treated mice. These incongruent results could be due to the age of the animals used (young growing versus mature adults), the species employed (rats versus mice), and/or the duration of the voluntary running protocol. Moreover, tamoxifen-treated Pax7/DTA mice, which were successfully depleted of Pax7+ cells (≤90 %), showed no increase in satellite cell content following 8 weeks of wheel running, indicating that this form of exercise is not a stimulus for satellite cell repletion.

The most surprising result of our study was that satellite cell-depleted mice ran markedly less than mice with the full complement of satellite cells; interestingly, the reduction in the distance run by satellite cell-depleted animals was mostly driven by a reduction in running speed, not the average time that the animals spent on the wheels. Hypothesizing that this reduction in running performance was driven by an attenuation of fiber-type transitioning to less fatigue resistant MyHC isoforms with exercise, plantaris muscles were evaluated for MyHC expression. Plantaris muscles from running mice displayed a reduction in fast-twitch glycolytic fibers (MyHC IIb) and an accumulation of fast-twitch oxidative fibers (MyHC IIa) independent of satellite cell depletion, showing that the observed running performance decrements were not due to impaired fiber-type conversions and that fiber-type switching does not require the presence of satellite cells. These findings are consistent with other studies from our lab using the Pax7/DTA model that concluded that both the overload-induced fiber-type shift [15, 27] and the gradual fiber-type transition observed in aged mice [16] remain intact despite satellite cell depletion.

Satellite cells have long been thought to be virtually quiescent under resting conditions, but recent research suggests that they can regulate their environment through communication and/or close contact with other cell types, including CD31^+^ endothelial cells [34, 35]. Moreover, satellite cells are able to connect with neighboring adult myofibers via ultrafine membrane structures named tunneling nanotubes [36] allowing for the transfer of cytoplasmic material, even organelles such as mitochondria. Therefore, we investigated whether changes in mitochondrial and/or capillary density that are positively associated with aerobic activity [12, 37] were dysregulated in the absence of satellite cells, potentially explaining the decrease in running performance. However, both SDH activity and VDAC abundance were not altered with satellite cell depletion, indicating that the presence of satellite cells did not affect mitochondrial function and/or content. Similarly, CD31+ (endothelial) cells and Oil Red O content were found to be increased in plantaris muscles from running mice, independent of the presence, or absence, of satellite cells. Therefore, we conclude that these adaptations to aerobic exercise are not coupled to satellite cell content and consequently cannot explain the observed decrement in running performance in satellite cell-depleted mice.

Recent work in our laboratory [15] and others [8, 38] has shown a regulatory relationship between satellite cells and fibroblasts. Following 8 weeks of compensatory overload in Pax7/DTA mice, plantaris muscles from satellite cell-depleted animals exhibited blunted muscle hypertrophy and reduced whole muscle function [15]. This maladaptation to overload was attributed to marked increases in extracellular matrix deposition in satellite cell-depleted muscles leading to a more fibrotic muscle environment. Voluntary unloaded wheel running is not a hypertrophic stimulus, and consequently, there were no increases in mean fiber size, or plantaris muscle wet weight. Additionally, in contrast to the hypertrophic stimulus elicited in the compensatory overload model, there were no increases in extracellular matrix deposition, as measured by WGA staining, with wheel running regardless of satellite cell content. The non-injurious and low resistance nature of the model may not be a sufficient stimulus to drive muscle remodeling and extracellular matrix modifications. Whether loaded wheel running, which does increase muscle size in hind limb muscles [14, 31, 39], would increase fibrosis in satellite cell-depleted muscles remains to be determined.

There are two other potential reasons for the decrease in running activity, and we addressed both of these possibilities experimentally using different mouse models. First, an unintended central nervous system component to our experimental model could have been introduced by depleting Pax7+ cells in brain tissue, as there is a well-documented presence of Pax7+ nuclei in the central nervous system [40, 41]. We tested tamoxifen-induced recombination specifically in the brain by using the Pax7/ZsGreen reporter mouse in which Pax7-expressing nuclei express GFP upon tamoxifen administration. Pax7+ nuclei were detected by immunohistochemistry in both brain and muscles from vehicle- and tamoxifen-treated animals, but tamoxifen did not induce recombination in the brain, since green nuclei were only detected in muscles following tamoxifen treatment. In addition, Pax7/DTA mice treated with tamoxifen are not depleted of Pax7+ cells in the brain, indicating that the decrease in running performance is not caused by a central nervous system-mediated effect in our model. Second, the toxicity of tamoxifen and/or Cre-recombinase could have potentially decreased running performance. However, the Pax7CreER parental strain treated with tamoxifen did not exhibit decreased running performance as was observed in Pax7/DTA mice, indicating that it was not a side effect of tamoxifen treatment that caused the decrease in the distance run in satellite cell-depleted mice.

In the absence of any detectable changes in muscle fiber morphology, fiber-type composition, or markers of aerobic capacity and/or oxygen delivery, it became evident that additional functional assessments of satellite cell-depleted animals beyond wheel running were necessary to further investigate potential causes for the observed decrements in running performance. A battery of functional tests was performed to assess strength, gait, balance, and coordination on both vehicle- and tamoxifen-treated mice. Consistent with running performance, satellite cell-depleted mice performed considerably worse overall than non-depleted animals on all tests. In satellite cell-depleted animals, grip strength and measures of balance and coordination were markedly decreased and gait analysis indicated inferior posture and gait coordination. Previously published work from our laboratory showed no indication of force decrements in muscle fibers from satellite cell-depleted mice [10, 15], and therefore, the impaired coordination is not due to decreases in muscle strength in the satellite cell depleted mice.

The observed phenotype is very similar to that of Egr3 knockout mice [26] which lack functional proprioception in the form of muscle spindles. Spindle fibers are proprioceptors involved in motor control that contain both sensory and motor neuron innervations [42]. Each muscle spindle is encased in a connective tissue capsule which contains small specialized intrafusal fibers that run parallel to the much larger extrafusal fibers [42, 43]. It is well documented that intrafusal fibers contain Pax7+ cells [44]. Furthermore, it has recently been reported that intrafusal fibers actually have a higher density of Pax7+ cells than extrafusal fibers, highlighting the likely importance of satellite cells in spindle function [43]. We show that Pax7+ cells were present in muscle spindles from vehicle-treated animals but were notably absent in tamoxifen-treated animals. It was also noted that muscle spindles in tamoxifen-treated mice were less well-organized. Recognizing the importance of spindle fibers in communicating muscle orientation to the central nervous system via the afferent nervous system, it seems plausible that satellite cell ablation has a much more profound effect on spindle fiber function, than whole muscle function. Our results showing higher ECM deposition and intrafusal fiber atrophy in tamoxifen-treated mice indicate the possibility that the connective tissue surrounding muscle spindle fibers becomes dysregulated in the absence of satellite cells, thereby inhibiting proprioceptive function, as evidenced by a decrease in firing frequency. The increase in ECM deposition is most likely caused by the loss of interplay between satellite cells and fibroblasts, which we have previously observed [8, 15]. Capsular thickening and atrophy of distal intrafusal fibers are also evident in spindles from advanced murine muscle dystrophy [45], further supporting the idea that in models of satellite cell depletion, spindle fiber dysfunction may result.

## Conclusions

Contrary to the original hypothesis of the study, satellite cell depletion did not result in a lack of muscle adaptability in response to aerobic exercise: mitochondrial content, capillary density, and fiber-type composition showed appropriate adaptation to wheel running independent of satellite cell content. However, considerable decrements in running performance, coordination, strength, and balance were apparent in satellite cell-depleted mice, indicating that satellite cells have a yet unexplored role in muscle function. Our data show that muscle spindles are negatively impacted by satellite cell ablation with regards to ECM deposition and intrafusal fiber size, as well as function. Given the high density of Pax7+ cells within muscle spindles, we propose that satellite cells play an important role in muscle spindle function and their absence causes alterations in proprioception and suggest that future studies should be directed toward investigating this novel function.

## Additional files

Additional file 1:
**The peak rate of wheel rotation was significantly lower in satellite cell-depleted animals.** Adult vehicle or tamoxifen-treated female Pax7/DTA mice were given open access to running wheels for 8 weeks. A mechanical counter was used to record wheel rotations and was analyzed for peak running rate (counts/min) and running bout length (min). The animals had free access to food and water ad libitum and were checked daily for health and wellness. **(A)** The peak rate of wheel rotation was significantly lower in satellite-depleted animals. **(B)** When the duration of running was broken down into bouts, defined as periods of running with more than an 18 min break in between them, satellite cell-depleted mice tended to run for shorter bouts (147.8 ± 11.8 vs. 120.6 ± 8.4 min; *P* = 0.24) Black bars represent vehicle-treated animals, and gray bars represent tamoxifen-treated animals. *****indicates a significant effect of tamoxifen. Values are means ± SE. Significance was set at *p* ≤ 0.05. (PPTX 50 kb) Additional file 2:
**Tamoxifen alone does not cause running performance decrements.** To control for any potential adverse effects of tamoxifen, the parental strain Pax7 ^*CreER/CreER*^ was used as a treatment control. Adult (6 months) female Pax7 ^*CreER/CreER*^ (*n* = 6 per group) received either an intraperitoneal injection of tamoxifen at a dose of 2.5 mg/day for five consecutive days or were injected with a vehicle control (15 % ethanol in sunflower seed oil), followed by an 8-week washout period. The Pax7 ^*CreER/CreER*^ mice ran for 6 weeks prior to sacrifice to assess the independent effects of tamoxifen on running performance. Running distance is reported as (km/day) and is represented temporally over the 6-week running period. Significance was set at *p* ≤ 0.05. (PPTX 47 kb) Additional file 3:
**No significant changes in muscle weights following 8 weeks of voluntary wheel running.** Plantar Flexor muscles (plantaris, gastrocnemius and soleus muscles) were weighed immediately after dissection and presented both as absolute muscle wet weight (mg) and muscle wet weight relative to body weight (mg/g). Values are means ± SE. Significance was set at *p* ≤ 0.05. (PPTX 50 kb)

## Abbreviations

CD31: endothelial cell marker
CSA: cross-sectional area
DAPI: 4′,6-diamidino-2-phenylindole
ECM: extracellular matrix
HRP: horseradish peroxidase
IHC: immunohistochemistry
MyHC: myosin heavy chain
Pax7: paired box 7
PBS: phosphate-buffered saline
PFA: paraformaldehyde
RIPA: radioimmunoprecipitation assay
SDH: succinate dehydrogenase
TSA: tyramide signal amplification
VDAC: voltage dependent anion channel
WGA: wheat germ agglutinin
