# Supplementary figures and images for: Reduced voluntary running performance is associated with impaired coordination as a result of muscle satellite cell depletion in adult mice
Source: Skelet Muscle. 2015 Nov 16;5:41. doi: 10.1186/s13395-015-0065-3 (PMC4647638; doi:10.1186/s13395-015-0065-3)

## Slide 1
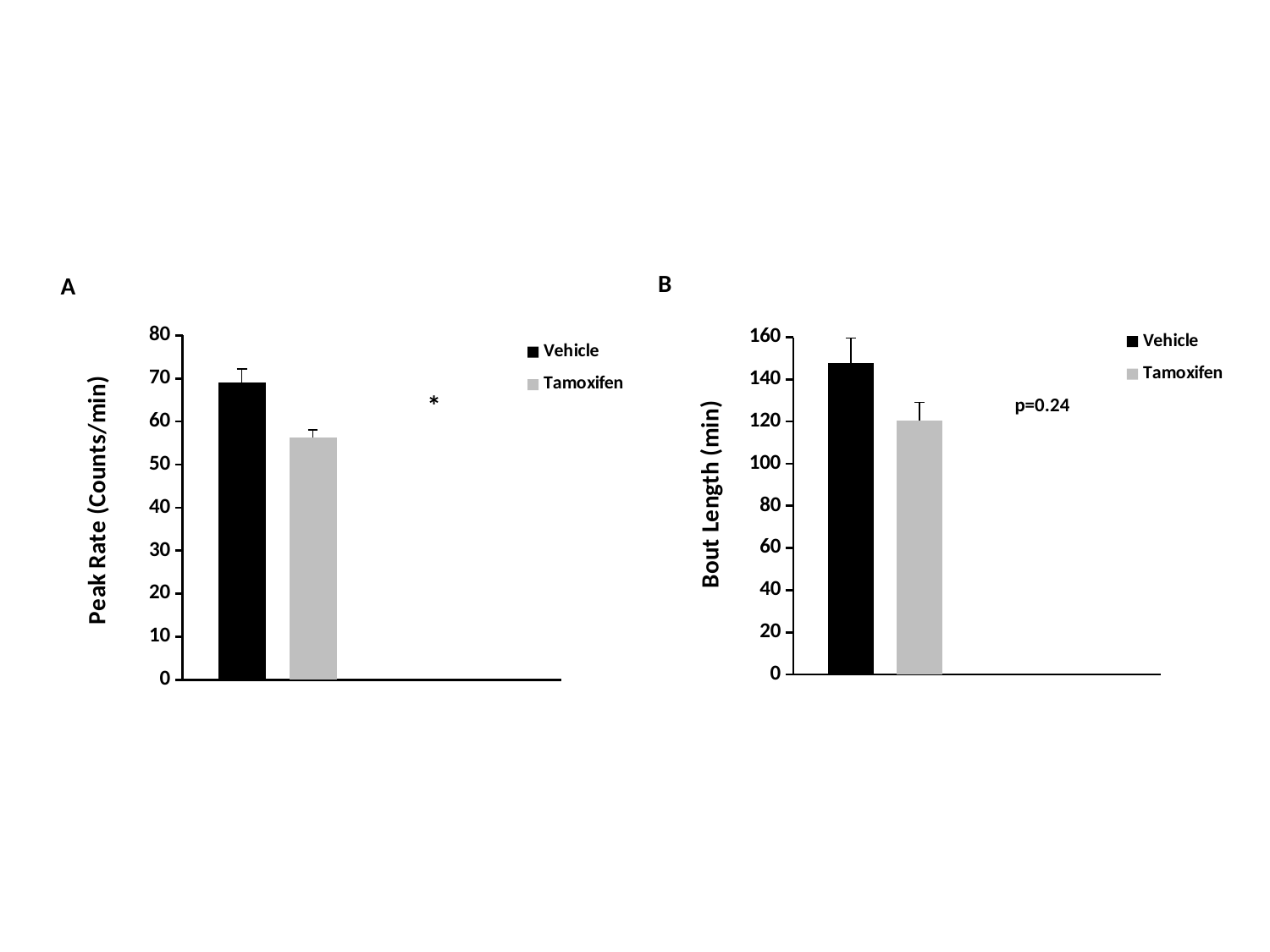

B
A
[unsupported chart]
p=0.24
[unsupported chart]
*

Supplement: Additional file 1: — The peak rate of wheel rotation was significantly lower in satellite cell-depleted animals. Adult vehicle or tamoxifen-treated female Pax7/DTA mice were given open access to running wheels for 8 weeks. A mechanical counter was used to record wheel rotations and was analyzed for peak running rate (counts/min) and running bout length (min). The animals had free access to food and water ad libitum and were checked daily for health and wellness. (A) The peak rate of wheel rotation was significantly lower in satellite-depleted animals. (B) When the duration of running was broken down into bouts, defined as periods of running with more than an 18 min break in between them, satellite cell-depleted mice tended to run for shorter bouts (147.8 ± 11.8 vs. 120.6 ± 8.4 min; P = 0.24) Black bars represent vehicle-treated animals, and gray bars represent tamoxifen-treated animals. *indicates a significant effect of tamoxifen. Values are means ± SE. Significance was set at p ≤ 0.05. (PPTX 50 kb) [file 13395_2015_65_MOESM1_ESM.pptx]

## Slide 1
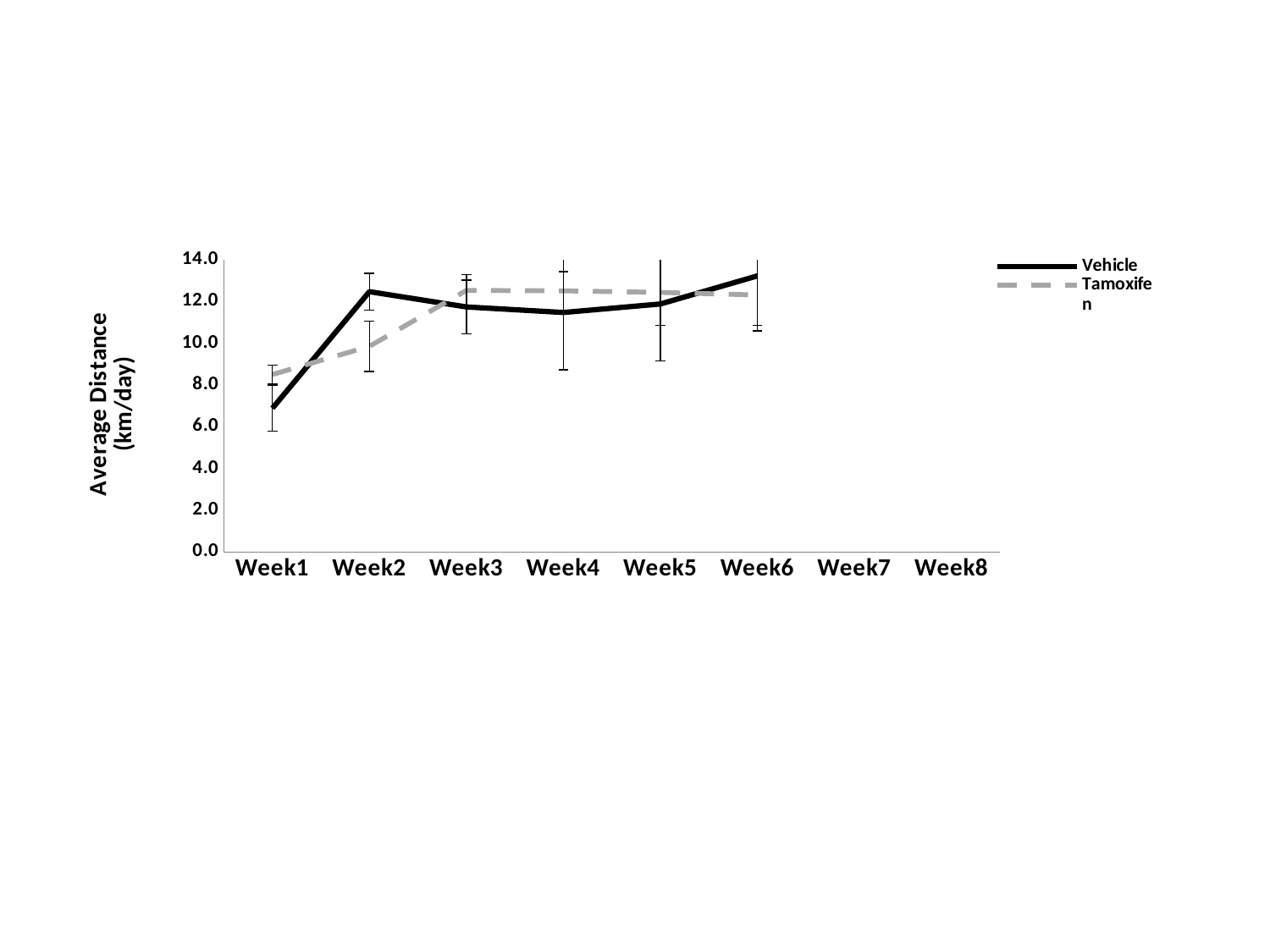

[unsupported chart]

Supplement: Additional file 2: — Tamoxifen alone does not cause running performance decrements. To control for any potential adverse effects of tamoxifen, the parental strain Pax7 CreER/CreER was used as a treatment control. Adult (6 months) female Pax7 CreER/CreER (n = 6 per group) received either an intraperitoneal injection of tamoxifen at a dose of 2.5 mg/day for five consecutive days or were injected with a vehicle control (15 % ethanol in sunflower seed oil), followed by an 8-week washout period. The Pax7 CreER/CreER mice ran for 6 weeks prior to sacrifice to assess the independent effects of tamoxifen on running performance. Running distance is reported as (km/day) and is represented temporally over the 6-week running period. Significance was set at p ≤ 0.05. (PPTX 47 kb) [file 13395_2015_65_MOESM2_ESM.pptx]
