# Supplementary material for: Reduced voluntary running performance is associated with impaired coordination as a result of muscle satellite cell depletion in adult mice
Source: Skelet Muscle. 2015 Nov 16;5:41. doi: 10.1186/s13395-015-0065-3 (PMC4647638; doi:10.1186/s13395-015-0065-3)
Supplement: Additional file 3: — No significant changes in muscle weights following 8 weeks of voluntary wheel running. Plantar Flexor muscles (plantaris, gastrocnemius and soleus muscles) were weighed immediately after dissection and presented both as absolute muscle wet weight (mg) and muscle wet weight relative to body weight (mg/g). Values are means ± SE. Significance was set at p ≤ 0.05. (PPTX 50 kb) [file 13395_2015_65_MOESM3_ESM.pptx]

## Slide 1
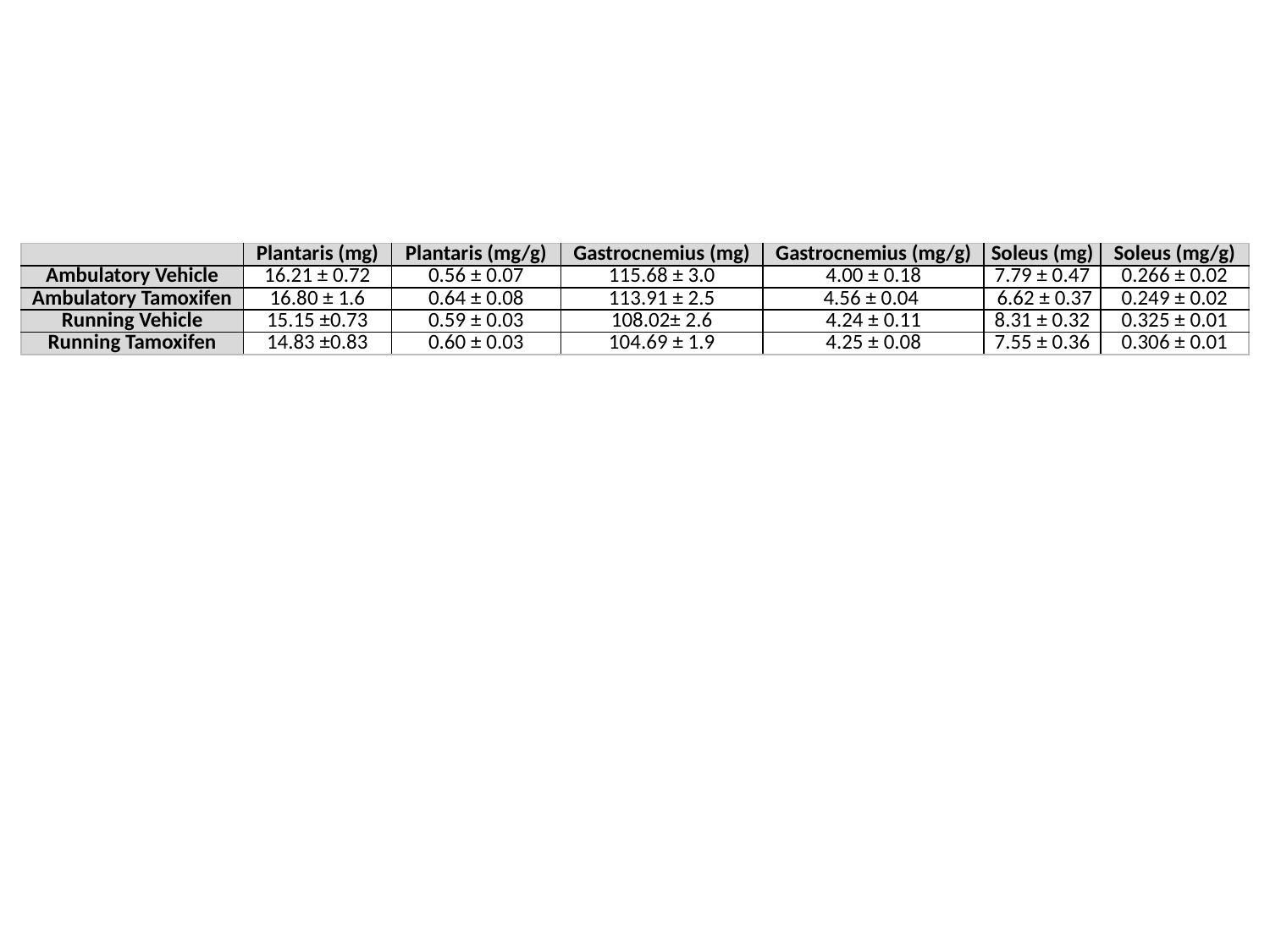

| | Plantaris (mg) | Plantaris (mg/g) | Gastrocnemius (mg) | Gastrocnemius (mg/g) | Soleus (mg) | Soleus (mg/g) |
| --- | --- | --- | --- | --- | --- | --- |
| Ambulatory Vehicle | 16.21 ± 0.72 | 0.56 ± 0.07 | 115.68 ± 3.0 | 4.00 ± 0.18 | 7.79 ± 0.47 | 0.266 ± 0.02 |
| Ambulatory Tamoxifen | 16.80 ± 1.6 | 0.64 ± 0.08 | 113.91 ± 2.5 | 4.56 ± 0.04 | 6.62 ± 0.37 | 0.249 ± 0.02 |
| Running Vehicle | 15.15 ±0.73 | 0.59 ± 0.03 | 108.02± 2.6 | 4.24 ± 0.11 | 8.31 ± 0.32 | 0.325 ± 0.01 |
| Running Tamoxifen | 14.83 ±0.83 | 0.60 ± 0.03 | 104.69 ± 1.9 | 4.25 ± 0.08 | 7.55 ± 0.36 | 0.306 ± 0.01 |
